# Paralysie faciale périphérique de l´enfant: une manifestation inhabituelle d´un corps étranger de l´oreille

**DOI:** 10.11604/pamj.2020.36.284.24308

**Published:** 2020-08-17

**Authors:** Patrick Maholisoa Randrianandraina, Hery Henintsoa Randrianirina, Avisoa Theodora Fare, Ando Mathieu Andriamahenina, Ravaka Hariniaina Andriambelo, Diavolana Koecher Andrianarimanana, Fanomezantsoa Andriamparany Rakoto

**Affiliations:** 1 Service d´Oto-Rhino-Laryngologie et Chirurgie Cervico-Faciale, Centre Hospitalier Universitaire Professeur Zafisaona Gabriel, Mahajanga, Madagascar,; 2 Service d´Anesthésie-Réanimation, Centre Hospitalier Universitaire Professeur Zafisaona Gabriel, Mahajanga, Madagascar,; 3 Service d´Ophtalmologie, Centre Hospitalier Universitaire Professeur Zafisaona Gabriel, Mahajanga, Madagascar,; 4 Faculté de Médecine, Université de Mahajanga, Mahajanga, Madagascar,; 5 Faculté de Médecine, Université d´Antananarivo, Antananarivo, Madagascar

**Keywords:** Abrus precatorius, corps étrangers, méat acoustique externe, paralysie faciale périphérique, Abrus precatorius, foreign bodies, external auditory canal, peripheral facial palsy

## Abstract

Les corps étrangers de l´oreille, fréquents chez l´enfant, se compliquent rarement. Nous rapportons un cas rare de paralysie faciale de l´enfant compliquant un corps étranger toxique de graine d´Abrus precatorius. Il s´agissait d´une enfant de 4 ans qui avait introduit ce type de corps étranger dans l´oreille, ayant entrainé, 15 jours plus tard une paralysie faciale homolatérale. Des signes de nécroses locaux associés à une otite externe étaient retrouvés sans atteinte systémique. L´extraction du corps étranger était effectuée au micro crochet. Des traitements locaux et généraux ainsi qu´une corticothérapie et une kinésithérapie faciale avaient permis une bonne évolution de la paralysie faciale au bout de trois semaines. Le séjour prolongé du corps étranger dans l´oreille et l´infection locale qui en résulte favorisent la survenue des complications fonctionnelles. L´extraction précoce et les soins locaux constituent les bases de leur traitement.

## Abstract (en)

Foreign bodies in the ear are common in children but they rarely lead to complications. We here report a rare case of homolateral facial palsy in a 4-year old female child that occurred 15 days after introduction of toxic foreign body (Abrus precatorius seed) in her ear. The patients had signs of local necrosis associated with external otitis, without systemic involvement. Foreign body extraction was performed using micro-hook. Local and general treatments as well as corticosteroid therapy and physiotherapy were started with good outcome after three weeks. Prolonged stay of the foreign body in the ear can cause local infection resulting in potential functional complications. Early extraction and local treatment are the basis for treatment.

## Introduction

La paralysie faciale entraine un préjudice esthétique, responsable de conséquences psychosociales importantes. La connaissance de ses étiologies permet de préciser la prise en charge et le pronostic de cette pathologie. La paralysie faciale de l´enfant peut être congénitale, traumatique à la naissance, développementale ou acquise [1]. Les étiologies des paralysies faciales acquises de l´enfant diffèrent peu de celles de l´adulte dont la plus fréquente est la paralysie de Bell. Cependant, les corps étrangers de l´oreille de l´enfant ont rarement été rapportés dans ce panel d´étiologies [1, 2]. Particulièrement fréquents chez l´enfant, ces corps étrangers peuvent générer des séquelles anatomiques et fonctionnelles loco régionales importants [3], justifiant une attention particulière et une prise en charge urgente. Nous rapportons un cas de paralysie faciale périphérique de l´enfant, suite à un corps étranger toxique de l´oreille de type végétal, chez un enfant malgache.

## Patient et observation

**Figure 1:**
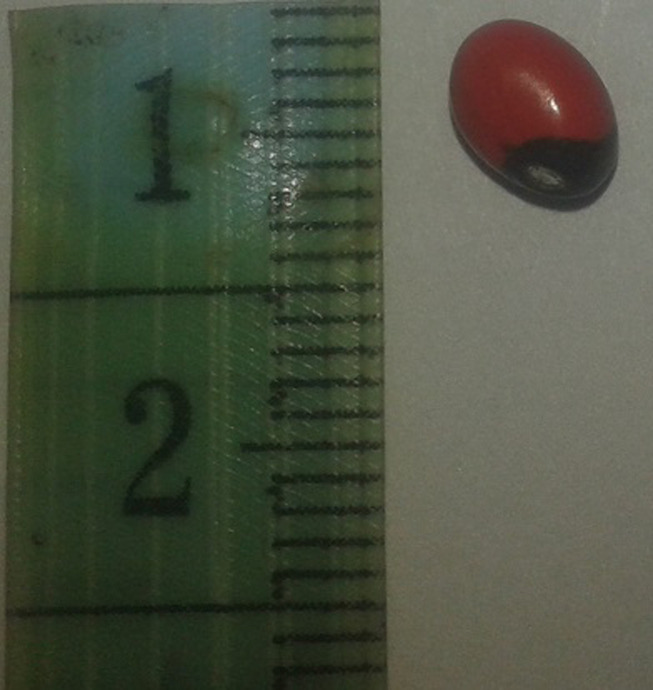
photographie d´une graine d´*Abrus precatorius*

Une fillette de 4 ans, habitante d'une commune rurale enclavée à une centaine de kilomètres de Mahajanga, était emmenée en consultation au service d´Oto-rhino-laryngologie du CHU Professeur Zafisaona Gabriel Mahajanga, pour une otalgie droite associée à une asymétrie du visage. Cette enfant sans antécédents particuliers, avait introduit une graine d´*Abrus precatorius* dans l´oreille droite, 15 jours avant la consultation. Deux échecs de tentatives d´extraction ont été effectués dans la formation sanitaire locale. Une otalgie droite au 10ème jour, suivie d´une otorrhée purulente et d´une asymétrie du visage d´installation progressive à partir du 14^e^jour ont motivé la consultation en ORL à Mahajanga. A l´examen, l´état général était conservé. Une paralysie faciale périphérique droite, gradée 5 dans la classification de House et Brackmann était objectivée (Figure 1). Elle était associée à une hypoacousie droite sans vertige ni trouble de l´équilibre et d´une otorrhée purulente droite. L´otoscopie droite retrouvait un corps étranger de type végétale enclavée et obstruant totalement le méat acoustique externe droit. La peau du méat acoustique externe était inflammée et œdématiée, il n´y avait pas d´éruptions cutanées locales. Le tympan gauche était normal. Il n´y avait pas de signe de méningite et le reste de l´examen ORL et général étaient normaux.

**Figure 2:**
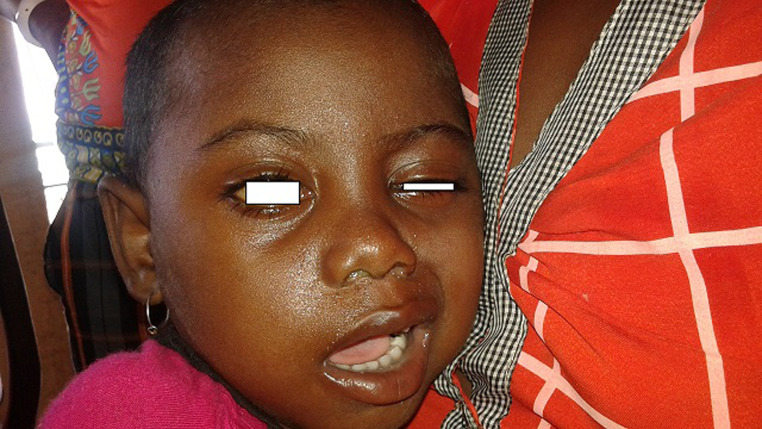
paralysie faciale sévère, grade 5 de House et Brackmann chez le cas présent

**Figure 3:**
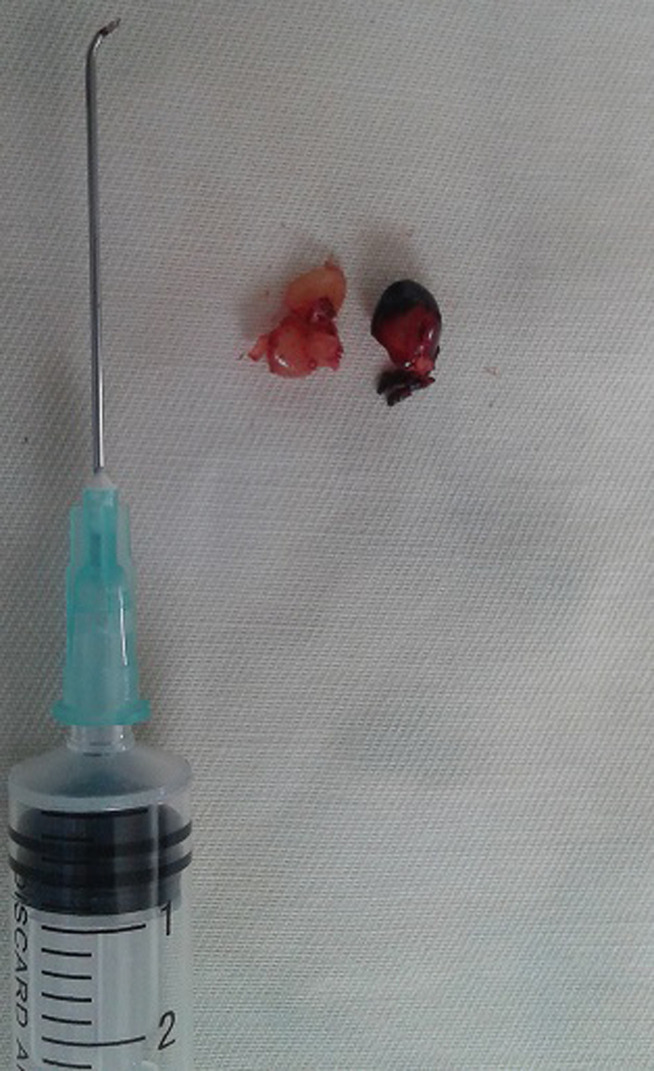
graine d´*Abrus Precatorius* extraite en 2 morceaux par un microcrochet confectionné avec une aiguille

Le bilan sanguin montrait une hyperleucocytose à polynucléaires neutrophiles, le dosage de la proteine-C réactive était à 24 milligrammes /litres, les bilans hépatiques et rénaux étaient normaux ainsi que la glycémie. Le prélèvement d´otorrhée retrouvait un *pseudomonas aeruginosa* sensible à la ciprofloxacine et des anaérobies. L´audiométrie tonale retrouvait une surdité de transmission droite avec une audition normale à gauche (Figure 2). La prise en charge immédiate était constituée par l´extraction du corps étranger avec un micro crochet confectionné à partir d´une aiguille (Figure 3), sous anesthésie générale. A l´issue de l´extraction, l´otoscopie retrouvait une perforation tympanique postérieure marginale, intéressant les 2/3 de la pars tensa, avec une nécrose cutanée du fond du méat acoustique externe. En plus d´antalgiques par voie orale, des soins locaux par aspiration quotidienne étaient associés à une antibiothérapie locale et générale, à base de ciprofloxacine et de métronidazole pendant 3 semaines. La paralysie faciale était traitée pendant 10 jours par la prednisolone à 1milligramme par kilogramme de poids par jour, des soins oculaires et une kinésithérapie faciale. A 3 semaines de traitement on notait la disparition totale de la douleur et une amélioration de la paralysie faciale (Grade 2 de House et Brackmann) avec un tarissement de l´otorrhée. Par contre la perforation tympanique et l´hypoacousie de transmission ont persisté. Un consentement éclairé pour publication anonyme du cas et des photos a été présenté et accepté par ses parents.

## Discussion

Les corps étrangers de la sphère ORL surviennent surtout chez l´enfant pendant l´âge de découverte et de préhension. La localisation auriculaire varie selon les auteurs de 14 à 68% des cas [4]. La plupart des complications des corps étrangers de l´oreille rapportés sont sans gravités. Ce sont des plaies du méat acoustique externe ou des otorragies résultantes de tentatives multiples d´extraction, ne nécessitant rarement des traitements [5]. Les complications graves les plus rapportées sont les perforations tympaniques, l´enclavement dans l´oreille moyenne [5] et la labyrinthite qui sont rares [6]. Nous rapportons un cas de corps étranger de l´oreille associé à une complication grave intéressant l´oreille moyenne et le nerf facial. La sévérité de ces complications dépend de deux paramètres principaux: la durée du séjour du corps étranger dans l´oreille [7] et la nature du corps étranger [4, 6]. Dans la majorité des cas, les corps étrangers de l´oreille sont asymptomatiques et souvent de découverte fortuites [6] responsable d´un retard de diagnostic, et donc d´un plus long séjour dans l´oreille. Les symptômes révélateurs peuvent être tardifs comme l´otalgie, l´otorrhée et l´hypoacousie [4] comme rapportés dans la présente observation. Le retard de prise en charge de 15 jours pourrait être en relation avec l´éloignement du domicile (100 kilomètres: 2 jours de marche et trajet fluvial) et la précarité des centres de soins locaux.

Pour leurs natures, les corps étrangers de l´oreille peuvent être organiques ou non. Les piles boutons sont les plus dangereux car ils libèrent des produits toxiques responsables de lésions nécrosantes au niveau de l´oreille [4, 8], nécessitant une extraction urgente. Les corps étrangers organiques entrainent une inflammation locale en activant le système immunitaire [8]. Dans le cas rapporté, le corps étranger était de type organique, une graine d´*Abrus precatorius*. Peu d´auteurs ont rapportés des cas d´atteinte locaux par ces graines toxiques. Cette graine provient d´une plante grimpante de type liane, présente en région tropicale comme Madagascar, dont les graines sont de couleur rouge écarlate avec une tache noire près du hile. Couramment utilisée comme antitussif ou comme ornements de colliers par les ménages de certaines régions de Madagascar, cette graine est facilement accessible par les enfants. Cependant, ils sont hautement toxiques par l´abrine qu´elles contiennent, pouvant être létale sans aucun antidote découvert [9]. L´abrine est une toxine qui inhibe la synthèse des protéines causant une destruction cellulaire par voie systémique. Chez des patients décédés de graines d´*Abrus precatorius* pillés ingérés, des lésions ulcérées de la muqueuse intestinale ont été rapportées ainsi que des signes neurologiques à type d´encéphalopathies, de convulsions et des tremblements [9]. Cependant peu d´études ont rapporté les effets locaux par contact direct de cette toxine. Chez l´enfant rapportée dans cette étude, aucun signe d´atteinte systémique ni de lésion viscérale n´ont été retrouvés. Les manifestations cliniques étaient surtout locales.

En plus, des lésions occasionnées par les tentatives d´extraction, les nécroses cutanées, la perforation tympanique et la lésion du nerf facial au niveau de son trajet derrière l´oreille moyenne pourrait correspondre à une toxicité locale de la graine d´*Abrus precatorius*. L´absence d´un scanner du rocher pour un bilan lésionnel ainsi qu´une étude des effets locaux de l´abrine limitaient l´explication de l´hypothèse de toxicité locale. Un corps étranger enclavé peut entraver le drainage naturel du méat acoustique externe. Ce qui est responsable d´une stase et d´une sensibilité accrue aux infections [6]. L´infection par *Pseudomonas aeruginosa*, a été constatée chez notre patiente. L´antibiothérapie locale adaptée serait ainsi nécessaire en cas de corps étranger de l´oreille. L´extraction des corps étrangers de l´oreille se fait soit sous anesthésie locale de Xylocaïne à 2% soit sous anesthésie générale si l´enfant n´est pas coopératif ou si plusieurs tentatives d´extraction ont échouées [4, 10]. Les techniques d´extraction proposées selon le type de corps étranger suspecté, la situation clinique et l´expérience du praticien sont l´extraction à la micro pince, au micro crochet, à l´aspiration et par le lavage d´oreille. Comme pour les piles boutons, le lavage d´oreille est à éviter en cas de corps étranger de graine d´*Abrus precatorius* car cette technique favoriserait la dissémination de la toxine sur les tissus environnants [10], mais entrainerait également un gonflement du corps étranger organique en contact avec l´eau, favorisant encore plus l´enclavement de ce dernier. L´extraction urgente du corps étranger toxique ainsi que l´aspiration régulière de la toxine au niveau de l´oreille, permettrait de contenir les effets néfastes de cette dernière. En plus de la corticothérapie précoce, le pronostic de récupération de la paralysie faciale chez l´enfant repose sur une kinésithérapie faciale [2], qui a été effective chez notre patiente. Une prise en charge précoce d´un corps étranger de l´oreille permettrait d´éviter ses complications.

## Conclusion

La paralysie faciale est une complication rare, à ne pas méconnaitre, d´un corps étranger de l´oreille chez l´enfant. Tout praticien devrait y penser surtout en cas de suspicion de corps étranger toxique car la proximité du nerf facial avec l´oreille l´expose à des lésions traumatiques. Nous rapportons le premier cas d´un corps étranger de graine d´*Abrus precatorius* dans l´oreille. C´est un type de corps étranger organique dangereux que l´on peut rencontrer en pays tropical, pouvant être responsable d´une nécrose locale de l´oreille. Son extraction est alors urgente. Comme tout corps étranger de l´oreille, la surveillance des enfants au moment des jeux est essentielle pour éviter l´accident, d´autant plus que la graine d´*Abrus precatorius* peut être mortelle en cas d´ingestion.
